# Transient Heat Waves May Affect the Photosynthetic Capacity of Susceptible Wheat Genotypes Due to Insufficient Photosystem I Photoprotection

**DOI:** 10.3390/plants8080282

**Published:** 2019-08-12

**Authors:** Erik Chovancek, Marek Zivcak, Lenka Botyanszka, Pavol Hauptvogel, Xinghong Yang, Svetlana Misheva, Sajad Hussain, Marian Brestic

**Affiliations:** 1 Department of Plant Physiology, Faculty of Agrobiology and Food Resources, Slovak University of Agriculture, Trieda A. Hlinku 2, 949 76 Nitra, Slovakia; 2 National Agricultural and Food Centre, Research Institute of Plant Production, Bratislavska cesta 122, 921 68 Piešt’any, Slovakia; 3 College of Life Science, State Key Laboratory of Crop Biology, Shandong Key Laboratory of Crop Biology, Shandong Agricultural University, Taian 271018, China; 4 Institute of Plant Physiology and Genetics, Bulgarian Academy of Sciences, 1113 Sofia, Bulgaria; 5 Key Laboratory of Crop Ecophysiology and Farming System in Southwest, Ministry of Agriculture, Sichuan Agricultural University, Chengdu 611130, China; 6 Department of Botany and Plant Physiology, Faculty of Agrobiology, Food and Natural Resources, Czech University of Life Sciences, 16500 Prague, Czech Republic

**Keywords:** high temperatures, heat stress, photosynthesis, photosystem I, photoprotection, photoinhibition, wheat

## Abstract

We assessed the photosynthetic responses of eight wheat varieties in conditions of a simulated heat wave in a transparent plastic tunnel for one week. We found that high temperatures (up to 38 °C at midday and above 20 °C at night) had a negative effect on the photosynthetic functions of the plants and provided differentiation of genotypes through sensitivity to heat. Measurements of gas exchange showed that the simulated heat wave led to a 40% decrease in photosynthetic activity on average in comparison to the control, with an unequal recovery of individual genotypes after a release from stress. Our results indicate that the ability to recover after heat stress was associated with an efficient regulation of linear electron transport and the prevention of over-reduction in the acceptor side of photosystem I.

## 1. Introduction

Climate change will bring about an increase in the frequency and intensity of weather extremes, such as heat waves and severe droughts [1,2]. Heat waves (high temperatures for a short time) can significantly reduce the production of grains [3]. Wheat (*Triticum aestivum* L.) is a major staple grain, with a global production of 772 million tons in 2017 [4]. To sustain or even increase production in the future for the rising needs of an increasing human population [5], ongoing adaptation in the form of breeding and suitable agronomic strategies is needed [4].

The optimum growing temperature for wheat is between 17 and 23 °C [6]. A plant is under heat stress when it is exposed to temperatures above an upper threshold for long enough to cause irreversible damage [7]. For wheat, threshold temperatures impacting growth and yield are between 31 and 35 °C [8,9,10], although some studies have reported high-temperature impacts above even 26 °C [11].

High temperatures cause protein denaturation and aggregation and increase the fluidity of membrane lipids. Indirect heat injuries comprise protein degradation, the inactivation of enzymes in the chloroplast and mitochondria, the inhibition of protein synthesis, and a loss of membrane integrity [12]. These injuries lead to the production of toxic compounds and reactive oxygen species (ROS), reduced ion flux, starvation, and the inhibition of growth [7]. Very high temperatures may cause cell death due to the collapse of cellular organization [13].

Increased temperatures typically lead to a reduction in stomatal conductance (*g_s_*) and thus closure of the stomata [14,15,16]. However, at high temperatures, *g_s_* might actually increase to avoid a lack of cooling and to avoid dangerously high leaf temperatures [17,18,19]. Stomatal conductance and net photosynthesis are inhibited by moderate heat stress in many plant species due to decreases in the activation state of rubisco [20,21].

The sites of photochemical reactions are among the first ones to be injured at high temperatures [22]. High temperatures can damage photosystem II (PSII), the oxygen-evolving complex (OEC), and electron transport at both the donor and acceptor sides of PSII in the photosynthetic apparatus [23,24,25,26]. PSII is not very stable at high temperatures, and its activity is reduced [27]. Heat stress may cause a dissociation of the OEC and thus an imbalance between the electron flow from the OEC toward the acceptor side of PSII [28].

Not all genotypes within a species have the same ability to cope with heat stress. There is a great deal of variation between and within species, providing opportunities to improve crop heat stress tolerance through genetic means [7]. However, to achieve this goal, contributions from plant physiologists, molecular biologists, and crop breeders are needed. The aim of this study was to provide physiological insights into the effects of a temporary heat wave on photosynthetic functions of wheat leaves, including recovery after heat stress. We focused on the diversity of responses in a group of diverse wheat genotypes of different origins in order to distinguish the photosynthetic responses associated with heat tolerance. 

## 2. Materials and Methods 

### 2.1. Cultivation of Plants

Eight cultivars of winter wheat (*Triticum aestivum* L.) (Equinox (origin: GBR), Thesee (FRA), 16/26 (SVK), GRC 867 (GRC), Roter Samtiger Kolb. (DEU), Unmedpur Mummy (EGY), Dušan (SRB), and AZESVK2009-90 (GEO)) were sown in the middle of November, cultivated at moderate temperature (10–15 °C) for approximately 1 month, and then vernalized in a growth chamber at 5 °C for a photoperiod of 12/12 h (light/dark) for four months, which is the typical duration of the winter period in Slovak wheat production areas. The plants were transplanted during the spring period (May) into pots with standard peat substrate and 5 g of Osmocote fertilizer. The plants were grown individually (one plant per pot) outdoors and were exposed to direct sunlight and natural climatic conditions. The pots were organized in a block with extra border plants, eliminating the effect of borders. The pots were irrigated regularly to prevent dehydration. The high-temperature treatment was started when all plants had fully developed spikes and flag leaves. 

### 2.2. Heat Wave Simulation and Measuring Protocol

The study was carried out at the Slovak University of Agriculture in Nitra, Slovakia. The heat wave was simulated by keeping the plants enclosed under a transparent polyethylene foil tunnel with a high light transmission (>90% of transmitted light at midday) starting in mid-June. Temperatures up to 38 °C were reached inside the tunnel, whereas outside temperatures were between 25 and 30 °C. The measurements were taken between 15 June and 27 June 2018; heat stress was measured on 18, 20, 21, and 22 June (T1 and T2 phase); controls (C) were measured on 19 and 26 June, and the recovery phase (R) was measured on 25 and 27 June. In the recovery phase, the heat-stressed plants were put in control conditions. The measurements of gas exchange and simultaneous measurements of photosystem I (PSI) and photosystem II (PSII) were taken in laboratory conditions.

### 2.3. Simultaneous Measurements of Gas Exchange and Chlorophyll Fluorescence

The measurements were carried out using an Li-6400 gasometer (LiCor, Lincoln, NE, USA) with simultaneous measurement of chlorophyll fluorescence [29]. The *F*_0_ and *F_m_* values were determined after 15 min of dark adaptation in a measuring head. Then, the sample was exposed to actinic light (1500 μmol photons m^−2^ s^−1^) at a leaf temperature of 25 °C with a reference CO_2_ content of 400 ppm and ambient air humidity. Every 2 min, the gas exchange rate was measured, followed by a saturation pulse and a far-red pulse, for *F_0_*′ determination. Then, a CO_2_ response curve was applied, starting with a record at 400 ppm and continuing with a stepwise change of levels of CO_2_: 300, 250, 200, 150, 100, 50, 400, 600, 800, 1000, 1200, and 1500. The values of gas exchange parameters (CO_2_ assimilation rate, *A*; stomatal conductance, *g_s_*; internal CO_2_ concentration, *Ci*) were calculated directly with a software gas analyzer. Calculations of the chlorophyll fluorescence (ChlF) parameters are described below. Further analyses of the A/Ci curves were performed using the Farquhar–von Caemerer–Berry model [30] edited by Ethier and Livingston [31]. 

### 2.4. Simultaneous Measurements of P700 Redox State and Chlorophyll Fluorescence

The state of PSI and PSII photochemistry was measured with a Dual PAM-100 (Walz, Effeltrich, Germany) with a ChlF unit and a P700 dual wavelength (830/875 nm) unit, as described by Klughammer and Schreiber [32]. Saturation pulses (10,000 μmol photons m^−2^ s^−1^), intended primarily for the determination of ChlF parameters, were also used for the assessment of P700 parameters. Prior to the measurements, the analyzed plants were dark-adapted. After determination of *F*_0_, *F_m_*, and *P_m_*, a moderate light intensity of 134 μmol photons m^−2^ s^−1^ was used to start up the photosynthetic process. After a steady state was reached, a rapid light curve was triggered (with light intensities of 14, 21, 30, 45, 61, 78, 103, 134, 174, 224, 281, 347, 438, 539, 668, 833, 1036, 1295, 1602, and 1960 μmol photons m^−2^ s^−1^ for 30 s at each light intensity). There was a saturation pulse and a far-red pulse for *F*′_0_ determination after 30 s at each light intensity. For the calculation of the ChlF parameters, the following basic values were used: *F*, *F*’, fluorescence emission from dark-or light-adapted leaf, respectively; *F*_0_, minimum fluorescence from dark-adapted leaf (PSII centers open); *F_m_*, *F′_m_*, maximum fluorescence from dark- or light-adapted leaf, respectively (PSII centers closed); *F*′_0_, minimum fluorescence from light-adapted leaf. The ChlF parameters were calculated as follows [33]: the maximum quantum yield of PSII photochemistry, *F_v_*/*F_m_* = (*F_m_* − *F*_0_)/*F_m_*; the actual quantum yield (efficiency) of PSII photochemistry, *Φ_PSII_* = (*F’_m_* − *F*′)/*F′_m_*; nonphotochemical quenching, *NPQ* = (*F_m_* − *F′_m_*)/*F′_m_*; the quantum efficiency of nonregulated energy dissipation in PSII, *Φ_NO_* = 1/(*NPQ* + 1 + *qL* (*F_m_*/*F_0_* − 1)); the quantum yield of pH-dependent energy dissipation in PSII, *Φ_NPQ_* = 1 − *Φ_PSII_* − *Φ_NO_*; and the redox poise of the primary electron acceptor of PSII, *Q_A_^−^*/*Q_A_* total = 1 − *qP*. The apparent electron transport rate of PSII photochemistry was calculated by assuming a leaf absorption of 0.84 and a PSII/PSI ratio of 1:1, *ETR_PSII_* = *Φ_PSII_* × *PAR* × 0.84 × 0.5.

For the calculation of the P700 parameters, the following basic values were used: *P*, P700 absorbance at a given light intensity; and *P_m_*, *P′_m_*, the maximum P700 signal measured using a saturation light pulse following short far-red pre-illumination in a dark- or light-adapted state. The P700 parameters were calculated as follows [32]: the effective quantum yield (efficiency) of PSI photochemistry at a given PAR, *Φ_PSI_* = (*P′_m_* − *P*)/*P_m_*; the oxidation status of the PSI donor side, i.e., the fraction of P700 oxidized in a given state, P700^+^/P700 total = *Φ_ND_* = *P*/*P_m_*; and the reduction status of the PSI acceptor side, i.e., the fraction of overall P700 oxidized in a given state by a saturation pulse due to a lack of electron acceptors, *Φ_NA_* = (*P_m_* − *P′_m_*)/*P_m_*. The apparent electron transport rate of the PSI photochemistry was calculated by assuming a leaf absorption of 0.84 and a PSII/PSI ratio of 1:1, *ETR_PSI_* = *Φ_PSI_* × *PAR* × 0.84 × 0.5.

### 2.5. Data Processing and Analysis

The statistical significance of differences was assessed using ANOVA, and post hoc comparisons were performed using Duncan’s multiple test (STATISTICA 10, StatSoft, Tulsa, OK, USA). The mean values ± standard error (SE) are presented. At least four plants of each of eight cultivars were measured through gas exchange and chlorophyll fluorescence measurements at four stages. The results of the statistical analyses are not indicated in the graphs, but all of the interpretations are based on these results. 

## 3. Results

**Figure 1:**
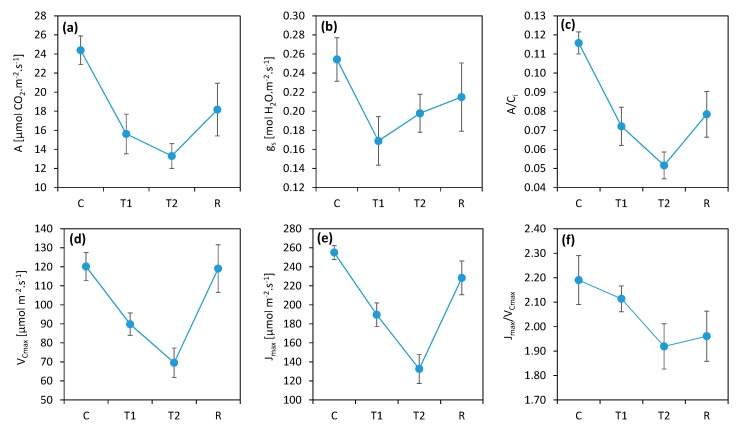
Heat effects on the parameters derived from the gas exchange measurements (**a**) A: photosynthesis rate; (**b**) *g*_s_: stomatal conductance; (**c**) *C_i_*: internal CO_2_ concentration; (**d**) *V_Cmax_*: maximum carboxylation rate; (**e**) *J_max_*: maximum electron transport velocity; (**f**) *J_max_*/*V_Cmax_* ratio. C: control; T1: thermal effect in the first phase; T2: thermal effect in the second phase; R: recovery phase. The points represent the mean values for all measured wheat plants of all genotypes. The error bars represent the standard error of the means.

The simulation of a heat wave by keeping the plants enclosed under a transparent foil tunnel was effective. The high temperatures (daily maximums at 38 °C and night minimums above 20 °C) had a significant negative effect on the photosynthetic functions of the plants and provided differentiation of genotypes by sensitivity to heat. The gas exchange measurements showed that the simulated heat wave led to a decrease in photosynthetic activity and stomatal conductance of 40% on average in comparison to the control, with a moderate recovery after the relief of stress (Figure 1). Plants that returned to normal conditions (R) after thermal stress showed persistent reductions in photosynthesis due to the several-day high-temperature periods. 

The ratio between the CO_2_ assimilation rate and the CO_2_ concentration in the intercellular spaces in the leaf (A/C_i_) expresses the efficiency of CO_2_ utilization by the photosynthetic apparatus and served to verify whether the monitored decrease in photosynthesis was caused by the closing of stomata or nonstomatal causes, mostly decreases in photosynthetic enzyme activities [29]. The decrease in A/C_i_ followed the trend of decreasing photosynthesis, which suggests that the closure of the stomata had only a marginal effect and that the nonstomatal limitation of photosynthesis was dominant. In addition, the trend of decreasing CO_2_ assimilation during the heat stress period (from T1 to T2) was opposite to that seen in stomatal conductance, suggesting a minor role of stomatal closure in the decrease in CO_2_ assimilation caused by the heat wave.

Further analysis of the A/C_i_ curves using the Farquhar–von Caemerer–Berry model [30] edited by Ethier and Livingston [31] helped reveal the partial limitations of the assimilation process. *V_Cmax_*, or maximum carboxylation rate, represented a limitation in rubisco enzyme activity, while *J_max_*, or maximum electron transport velocity, represented a limitation of the primary photosynthesis product in C3 plants, RuBP [34]. The results of these parameters corresponded quite well with photosynthesis, which means that they participated at the same proportion in the limitation of photosynthesis under the influence of heat. Thus, the photosynthetic limitation identified by the *A/C_i_* parameter was due both to a decrease in rubisco total activity and to a decrease in RuBP regeneration, which is usually attributed to limited electron transport in chloroplasts [30]. It is worth mentioning that the trend of *V_Cmax_* was not the same as *J_max_*. The maximum electron transport (*J_max_*) was more affected by heat stress compared to the carboxylation activity of rubisco (*V_Cmax_*) in conditions of a heat wave. Moreover, whereas *V_Cmax_* almost completely recovered, *J_max_* was still decreased after the heat wave. This trend was well illustrated by the *J_max_* to *V_Cmax_* ratio, which decreased due to heat stress, and no recovery was observed after heat stress ended. 

The high temperatures inflicted a nonstomatal limitation of photosynthesis. This effect was proven by the decrease in rubisco activity as well as by the parameters of photochemistry.

**Figure 2:**
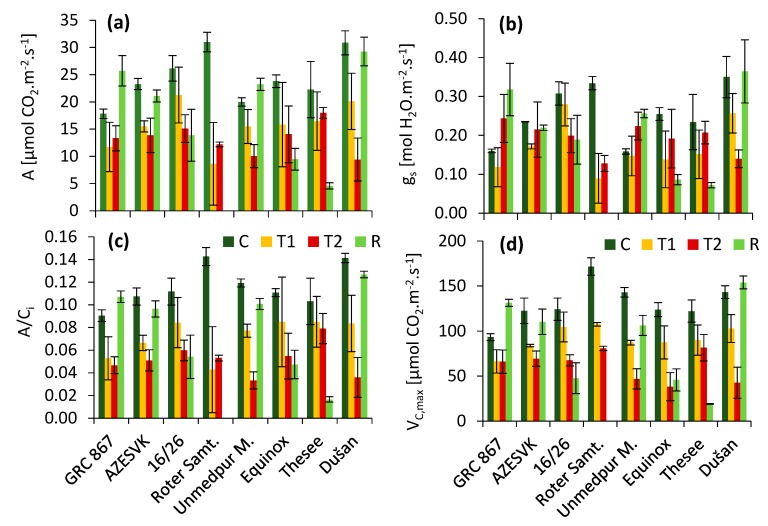
Heat effect on parameters measured by Li-6400 (**a**) A, photosynthesis rate; (**b**) *g_s_*, stomatal conductance; (**c**) A/C_i_, photosynthetic rate per unit of internal CO_2_ concentration; (**d**) *V_Cmax_*, maximum rate of carboxylation based on analyses of A/Ci curves; C, control; T1, thermal effect in the first phase; T2, thermal effect in the second phase; R, recovery phase. Mean values ± SE are presented.

By comparing different varieties (Figure 2), we found that there was considerable variability in the response to heat and in the ability to recover after stress. The highest photosynthesis levels were in the varieties Dušan and Roter Samtiger Kolbenweizen before the heat wave. At the same time, these were varieties that showed the highest rate of photosynthesis decrease after two (T1) and four (T2) days of temperature stress. These two varieties differed significantly in their ability to regenerate the photosynthetic apparatus. While the leaves of Roter Samtiger Kolbenweizen died rapidly after thermal stress (they did not regenerate), Dušan regenerated very well, similarly to genotypes GRC 867, AZESVK2009-90, and Unmedpur Mummy. The least influence of heat in phases T1 and T2 was observed in the variety GRC 867. The heat-stressed plants of genotype Thesee showed poor recovery and started premature senescence of the leaves, which did not occur in the control plants. Similar, but less evident, trends were also observed in cv. Equinox and 16/26. The values of stomatal conductance (*g_s_*) decreased, similarly to CO_2_ assimilation. However, the decrease in the *A/Ci* ratio indicated that stomata closure was not the major reason for the photosynthetic decline in stress and recovery conditions. Values for the maximum rate of carboxylation derived from the initial slope of the A/Ci curve (*V_Cmax_*) showed a very similar trend to the observed CO_2_ assimilation rate. 

**Figure 3:**
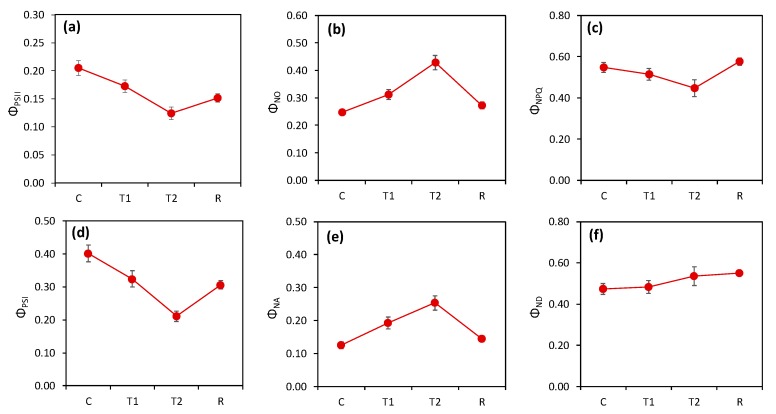
Heat effect on parameters measured by Dual-PAM (the average from all varieties): C, control; T1, thermal effect in the first phase; T2, thermal effect in the second phase; R, recovery phase. (**a**) The effective quantum yield of photosystem II (PSII) (*Ф_PSII_*); (**b**) the fraction of energy captured by PSII passively dissipated in the form of heat and fluorescence (*Ф_NO_*); (**c**) the quantum yield of regulated nonphotochemical quenching in PSII (*Ф_NPQ_*); (**d**) the effective quantum yield of PSI (*Ф_PSI_*); (**e**) the quantum yield of PSI nonphotochemical quenching caused by the acceptor side limitation, i.e., the fraction of overall P700 that could not be oxidized in a given state (*Ф_NA_*); (**f**) the quantum yield of PSI nonphotochemical quenching caused by the donor side limitation, i.e., the fraction of overall P700 that was oxidized in a given state (*Ф_ND_*). The points represent the mean values for all measured wheat plants of all genotypes. The error bars represent the standard error of the means.

The measurements of photosynthetic quantum yields of both photosystems showed a decrease in the activity of both photosystems in reaction to high temperatures. The high temperatures decreased the activity of both photosystems during stress as well as after stress. The heat simulated in the tunnel led to a decrease in the quantum efficiency of both PSII and PSI (Figure 3) by approximately 40–50%. The quantum yield of regulated nonphotochemical quenching (*Φ_NPQ_*) was lower in heat-stressed plants and, interestingly, higher in recovered plants than in the control. As a result of the decrease in *Φ_PSII_* and *Φ_NPQ_*, we observed very high values in the fraction of nonregulated (passive) nonphotochemical dissipation (*Φ_NO_*). The decrease in *Φ_PSI_* was caused by an increase in the acceptor side limitation (*Φ_NA_*). Interestingly, the values of the quantum yield of nonphotochemical quenching of PSI caused by the donor side limitation (*Φ_ND_*) did not change significantly.

**Figure 4:**
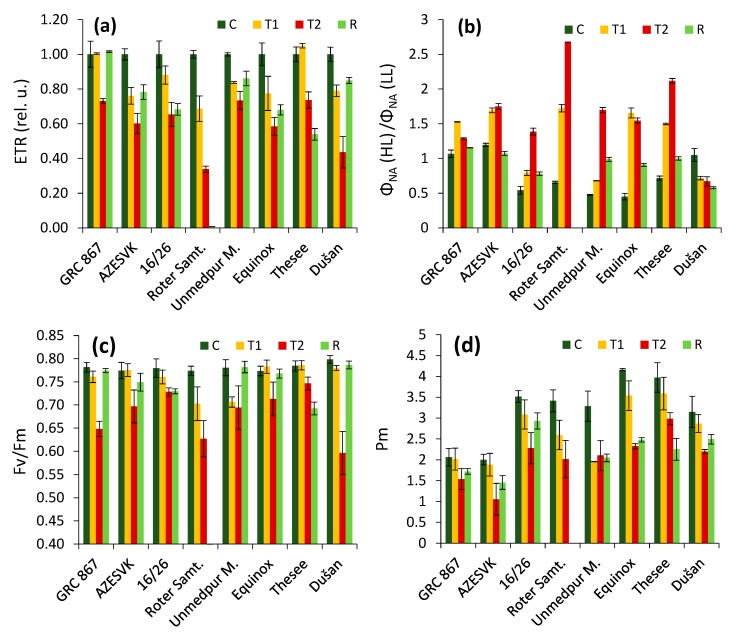
Effects of heat stress on parameters measured by Dual-PAM in different varieties: C, control; T1, thermal effect in the first phase; T2, thermal effect in the second phase; R, recovery phase. (**a**) The relative values of the electron transport rate (the average value of the control plants of each genotype equals 1). (**b**) The ratio of the acceptor side limitation measured in high light (HL, ~2000 µmol m^−2^ s^−1^) and the value measured in low light (LL, ~40 µmol m^−2^ s^−1^). (**c**) Maximum quantum efficiency of PSII photochemistry. (**d**) The maximum amplitude of P700 kinetics. Average values ± SE are presented.

The trends in the photosynthetic electron transport rate (ETR, Figure 4a) as well as the value of photosynthetic assimilation in different wheat genotypes (Figure 2) clearly indicated differences in the ability to recover after the heat stress period ended. The leaves of the genotype Roter Samtiger, especially, became necrotic and dried as a consequence of heat stress. Moreover, some other genotypes expressed insufficient recovery. We also analyzed how the genotypes were able to downregulate over-reduction in the PSI acceptor side (Figure 4b). We observed that the same genotypes were characterized by an over-reduction in the PSI acceptor side during the heat stress period, whereas this was not so obvious in well-recovering genotypes. These results were also evident in the values of fluorescence and the PSI parameters measured in a dark-adapted state. It was evident that the decrease in *F_v_/F_m_* was most severe in the Roter Samtiger cultivar and that the values were also not recovered in the Thesee cultivar. Different trends were observed in parameter *P_m_*, representing a maximum amplitude of P700 kinetics, in which we found only partial or no recovery (cv. Rotter Samtiger, Thesee) after relief from heat stress. Moreover, the genotypes differed in the severity of *P_m_* decrease and the level of *P_m_* recovery. 

**Figure 5:**
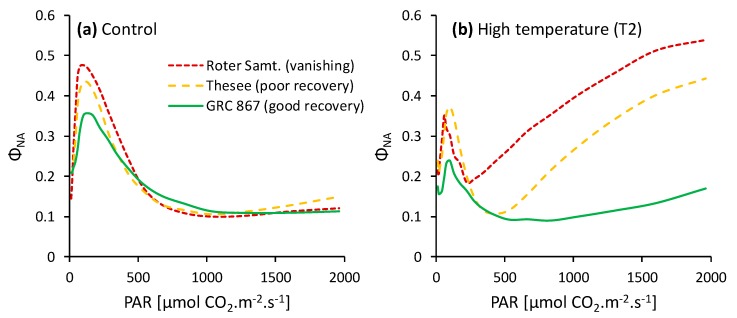
Examples of the light response curves of the PSI acceptor side limitation parameter (*Φ_NA_*) measured in control plants (**a**) and in plants exposed to the heat wave (**b**) of three genotypes differing in their capacity to recover after the withdrawal of heat stress.

Detailed analyses of the light response of the PSI acceptor side state (Figure 5) indicated that the well-regenerating genotype GRC 867 was able to efficiently downregulate electron transport, keeping the PSI acceptor side reduction low, even in very high light. The photoprotective capacity of genotypes became insufficient at light intensities over ~600 µmol m^−2^ s^−1^. The regulation of the electron transport in the genotype Roter Samtiger failed at a PAR below 300 µmol m^−2^ s^−1^. 

## 4. Discussion

There are several target sites for elevated temperature-induced damage, such as the CO_2_ fixation system, photophosphorylation, the electron transport chain, and the OEC [35]. The enzymes of the Calvin–Benson cycle are heat-labile. This means that the carbon assimilation system is sensitive to elevated temperatures and is strongly inhibited at moderate thermal stress [36]. These inhibiting effects are mostly observed when measured directly at high-temperature conditions [35,36], which was not the case in our experiments, in which photosynthesis was measured at a normal temperature (25 °C) at least 12 h after the last exposure to high temperature. The heat effects observed in our study were consequences and not the instantaneous effects of heat stress on photosynthesis. In this respect, it is interesting that the post-stress effects observed in our study were similar to the instantaneous effects well known from other studies. One of them was the decrease in rubisco activity indicated by the decrease in *V_Cmax_*, which was associated with a decrease in RuBP regeneration and the limitation in photosynthesis represented by the parameter *J_max_* (Figure 1). As direct damage to rubisco was not likely in the conditions of our experiments, an inhibition of the enzyme (especially rubisco activase) caused by its sensitivity to moderately high temperatures [37,38] is more probable. There is a hypothesis that a decrease in rubisco activation represents a protective mechanism against a critical decrease in the transthylakoid proton gradient in high-temperature conditions to prevent the collapse of photoprotective functions, with fatal consequences associated mainly with an uncontrolled increase of oxidative stress [36,39]. If long-term effects from high temperatures on photoprotection occur, the downregulation of enzyme activities might be needed even after temperatures return to normal. Moreover, the ratio of *J_max_* to *V_Cmax_* decreased due to stress (Figure 1f), which may indicate that electron transport-related processes were affected more than the carboxylation activity of rubisco was. Therefore, we focused on the processes associated with photosynthetic electron transport.

An analysis of basic chlorophyll fluorescence and P700 parameters in nonstressed plants (Figure 3) confirmed this expectation and identified the sustaining effects of high temperatures on PSII and PSI photochemistry, including the photoinhibition of PSI, which were similar to the effects observed in our previous study on wheat exposed to high temperature [40]. We previously showed that PSI photoinhibition had major effects on carbon assimilation [41] and photoprotection [42]. The trends in the parameters measured by simultaneous chlorophyll fluorescence and P700 (Figure 3) indirectly (e.g., a decrease in *Φ_NPQ_* despite a decrease in *Φ_PSII_*) or directly (increase in *Φ_NA_*) pointed to damage to PSI functions. Thus, our results support the hypothesis that the decrease in photosynthetic assimilation was associated with a decrease in photochemical activities.

In addition to the general trends, we observed some variance among the observed genotypes. The most important were the differences in recovery several days after heat stress relief, which was evident in the gas exchange as well as in the photochemical responses (Figure 2 and Figure 4). We observed an extreme response in genotype Roter Samtiger, in which heat stress led to severe necrosis and the death of leaves. In addition, we observed low recovery in some other genotypes, especially in the genotype Thesee. Interestingly, in these genotypes, we observed a very high level of over-reduction in the PSI acceptor side in high light conditions (high values of parameter *Φ_NA_*), whereas in well-regenerating genotypes, the parameter *Φ_NA_* was kept low in high light. It has previously been shown that a high *Φ_NA_* is an indicator of over-reduction in the PSI acceptor side [43,44,45,46], which leads to the excessive production of ROS in PSI [47,48,49]. Such a situation is known to be responsible for PSI photoinhibition in vivo [50,51]. PSI photoinhibition is characterized by very low recovery, and in some cases, PSI damage is not completely reversible [52,53]. Most photoinhibited PSI reaction center complexes are not repaired, but degrade after photoinhibition together with their binding chlorophylls [54]. This is a completely different situation compared to PSII, which is able to quickly recover. In the most sensitive genotypes, we observed a loss of ability to downregulate linear electron transport even at moderate light intensities, which might have easily resulted in leaf damage due to the accumulation of ROS in tissues. This explains the necrosis of leaves, which led to their premature death. A high ROS production could trigger the processes of early senescence associated with a decrease in the photosynthetic capacity of the leaves in sensitive genotypes. In the genotypes that had well-regulated electron transport, early senescence was not observed.

Considering the possible practical relevance of the *Φ_NA_* parameter measure during heat stress as an indicator of heat-sensitive genotypes, it must be noted that only the values of the *Φ_NA_* or *Φ_NA_* (HL) to *Φ_NA_* (LL) ratio (Figure 4) could not fully explain the level of recovery of photosynthetic capacity after the heat wave in all genotypes. For example, the values of the *Φ_NA_* (HL) to *Φ_NA_* (LL) ratio in the heat stress stage in cv. Equinox and AZESVK were similar, but AZESVK recovered better. In this respect, the changes in values in the *Φ_NA_* (HL) to *Φ_NA_* (LL) ratio between control conditions and heat wave conditions seemed to be more indicative. It is obvious that the sensitive cultivars showed a higher change in the values of the *Φ_NA_* (HL) to *Φ_NA_* (LL) ratio compared to the more resistant cultivars.

Rubisco activation, assessed by the initial slopes of the A/Ci curves [55], has previously been found to be a possible reason for the improper regulation of electron transport, identified at the level of PSI. When rubisco activity decreased, *Φ_NA_* increased, and *Φ_ND_* was suppressed [56]. The general trend of *Φ_NA_* increase (Figure 4 and Figure 5) could have been caused primarily by the difference in rubisco activation and a decrease in the need for an electron transport in photosynthesis. On the other hand, the differences in carboxylation activity observed in our study cannot explain the different trends of *Φ_NA_* shown in Figure 5. Therefore, we suggest that the different susceptibilities to high temperatures observed in our study were not directly associated with the rubisco activation state.

One interesting trend observed in our study was a higher decrease in the capacity of the electron transport rate (represented by *J_max_*) compared to the carboxylation capacity (*V_Cmax_*), both in heat stress and recovery periods. The values of *P_m_* corresponded better to the records of the CO_2_ assimilation rate or the ETR compared to *F_v_/F_m_* (Figure 4), especially during the recovery period. It is obvious that whereas PSII recovered very well (in six of eight genotypes), the recovery of PSI was much lower and was insufficient in most of the plants. Thus, the lower activity of PSI could have been responsible (at least partially) for the decrease in the electron transport capacity after the transient heat wave period.

Overall, the results suggest that the proper regulation of electron transport and the efficient photoprotection of PSI against photoinhibition were crucial in preventing negative post-stress effects after plants were exposed to short transient periods of high temperatures, which commonly occurs during the crop vegetative period. This may be important for breeding strategies, as the probability of heat waves will increase due to climate change.
